# Electroacupuncture alleviates chronic pain and comorbid depression through Gut-Metabolite-Brain axis remodeling to restore mPFC Glu/GABA balance

**DOI:** 10.1038/s41398-026-04544-y

**Published:** 2026-10-30

**Authors:** Sheng Li, Jianpeng Huang, Derong Lin, Ding Luo, Wenbin Fu, Jianhua Liu

**Affiliations:** 1 Acupuncture Research Team, the Second Affiliated Hospital of Guangzhou University of Chinese Medicine, Guangzhou, China; 2 Guangdong Provincial Key Laboratory of Acupuncture and moxibustion of Chinese Medicine, Guangzhou University of Chinese Medicine, Guangzhou, China; 3 Dongguan Hospital of Guangzhou University of Chinese Medicine, Dongguan, China

**Keywords:** Psychology, Psychiatric disorders

## Abstract

The comorbidity of chronic pain and depression is increasingly recognized as a systemic disorder involving dysregulated microbiota–gut–brain communication. Although electroacupuncture (EA) has demonstrated therapeutic efficacy for both pain and depression, the mechanisms linking peripheral microbial-metabolic remodeling to central neurotransmitter regulation remain incompletely understood. Here, we employed an integrated multi-omics approach in a spared nerve injury (SNI) rat model to investigate the gut–metabolite–brain mechanisms underlying EA’s efficacy. EA significantly alleviated mechanical hypersensitivity and depressive-like behaviors while restoring mPFC Glu/GABA balance, accompanied by normalization of the glutamate transporter EAAT2 and the GABA-synthesizing enzyme GAD67. Metagenomic analysis showed that EA remodeled the gut microbial ecosystem by increasing microbial diversity, suppressing opportunistic pathobionts, including *Bacillus cereus, Alistipes onderdonkii*, while enriching SCFA-associated genera, including *Butyricimonas and Romboutsia*. These alterations were accompanied by coordinated remodeling of amino acid and butanoate metabolism. Correlation network analysis identified Pipecolic acid as a central metabolic node linking gut microbial alterations with mPFC GABAergic neurotransmission and behavioral recovery. Although individual bacterial genera did not significantly mediate its restoration, causal mediation analysis demonstrated that the Pipecolic acid–GABA axis significantly mediated improvements in both mechanical allodynia and anhedonia, supporting Pipecolic acid as a functional metabolic hub linking peripheral metabolic remodeling to central GABAergic regulation. Collectively, these findings identify peripheral metabolic remodeling as a key mechanistic interface through which EA coordinates gut microbial ecology and central neurochemical homeostasis, providing a systems-level framework for understanding the therapeutic mechanisms of acupuncture in pain–depression comorbidity.

**Figure:**
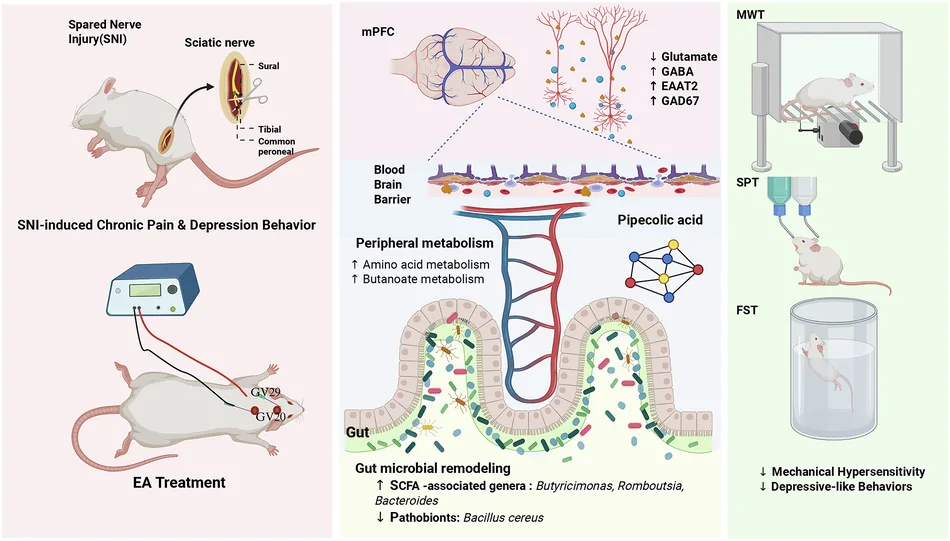

## Introduction

Chronic pain (CP) is a pervasive global health challenge affecting approximately 20% of the adult population, now recognized not merely as a symptom but as a distinct disease entity with complex biological, psychological, and social dimensions [1]. A critical clinical feature of CP is its high comorbidity with psychiatric disorders, particularly depression. Epidemiological studies indicate that 30% to 50% of patients with chronic pain suffer from co-occurring depression, a prevalence rate significantly higher than in the general population [2]. This ‘pain-depression dyad’ forms a vicious cycle of mutual exacerbation, significantly compromising prognosis and increasing disability. Current pharmacological management—relying heavily on opioids, non-steroidal anti-inflammatory drugs, and antidepressants—often yields suboptimal outcomes for this comorbidity and is frequently limited by tolerance, addiction risks, and systemic side effects [1, 3]. Consequently, there is a pressing demand for safe, effective, and well-tolerated non-pharmacological interventions.

The co-occurrence of chronic pain and depression is underpinned by shared neuroplastic maladaptations, particularly within the prefrontal cortex (PFC), a critical hub for pain modulation and emotional processing [4, 5]. It is well-established that the disruption of the PFC Glu/GABA balance precipitates the co-morbid pain and depressive behaviors [6, 7]. However, mounting evidence suggests that these central circuit disruptions are not an isolated event but are dynamically regulated via the bidirectional microbiota–gut–brain axis [8, 9]. Distinct signatures of gut dysbiosis have been consistently observed in both clinical patients and animal models of pain and depression [10, 11], and emerging research highlights that the gut microbiome possesses a profound capacity to modulate pain and depressive behaviors [12–14]. Mechanistically, gut microbiota communicates with the central nervous system through a bidirectional network utilizing neuroendocrine pathways, autonomic signaling, and host-microbial co-metabolites [15]. Thus, rebalancing the gut microbiota represents a promising therapeutic strategy for treating central nervous system disorders, including depression and chronic pain [16].

Electroacupuncture (EA) is currently recommended by multiple international clinical guidelines as a viable non-pharmacological treatment for chronic pain and depressive disorders [17, 18]. While classically attributed to central neuroplasticity modulation [19], recent investigations have increasingly focused on the gut-brain axis. Accumulating evidence indicates that EA can re-establish intestinal microbial homeostasis, which correlates with improved central neurotransmitter levels in various stress-related and neurological disorders [20, 21]. Consistent with these findings, our previous work demonstrated that EA alleviates SNI-induced comorbidities by modulating the gut microbiome structure and upregulating cerebral BDNF expression, a key regulator of neural plasticity [22]. However, these insights have been primarily limited to taxonomic composition (i.e., “who is there”), leaving the functional metabolic potential of the microbiome (i.e., “what they are doing”) largely unexplored. Crucially, the specific microbial metabolic functions and systemic mediators that physically bridge the gut-brain interaction remain elusive. Consequently, there is a paucity of integrated evidence determining whether EA ameliorates these deficits by mobilizing specific metabolic programs.

To provide such integrated evidence, we implemented a multi-omics approach combining fecal metagenomics and plasma untargeted metabolomics in a Spared Nerve Injury (SNI) rat model. This framework allowed us to map the systemic network alterations modulated by EA along the gut–metabolite–brain axis. Crucially, statistical mediation analysis identified a key peripheral-to-central signaling flow linking host–microbial co-metabolism to the regulation of mPFC Glu/GABA balance, revealing the mechanistic foundation for EA-induced recovery from co-morbid mechanical allodynia and depressive-like behaviors.

## Materials and methods

### Animals

A total of 24 specific-pathogen-free healthy, male Sprague Dawley rats (140–160 g) were purchased from Guangdong Medical Laboratory Center (Guangdong, China; No. 44829700021958; (SCXK) 2018-0094). Sample sizes were determined based on established published literature with consistent experimental endpoints, while fully complying with the 3Rs principle to minimize animal usage. No pre-defined inclusion or exclusion criteria were applied in this animal study. Rats were housed under a standard 12-hour light/dark cycle at a constant room temperature of 24 °C ± 1 °C with ad libitum access to food and water. All animal care and experimental procedures were according to the National Institutes of Health Guide for the Care and Use of Laboratory Animals and followed the guidelines of the Ethics Committee of Guangdong Provincial Hospital of Chinese Medicine, Guangdong Provincial Hospital of TCM (permit number: 2022032). A minimum acclimatization period of 1 week was allowed before commencement of experiments.

### Experimental design

**Fig. 1:**
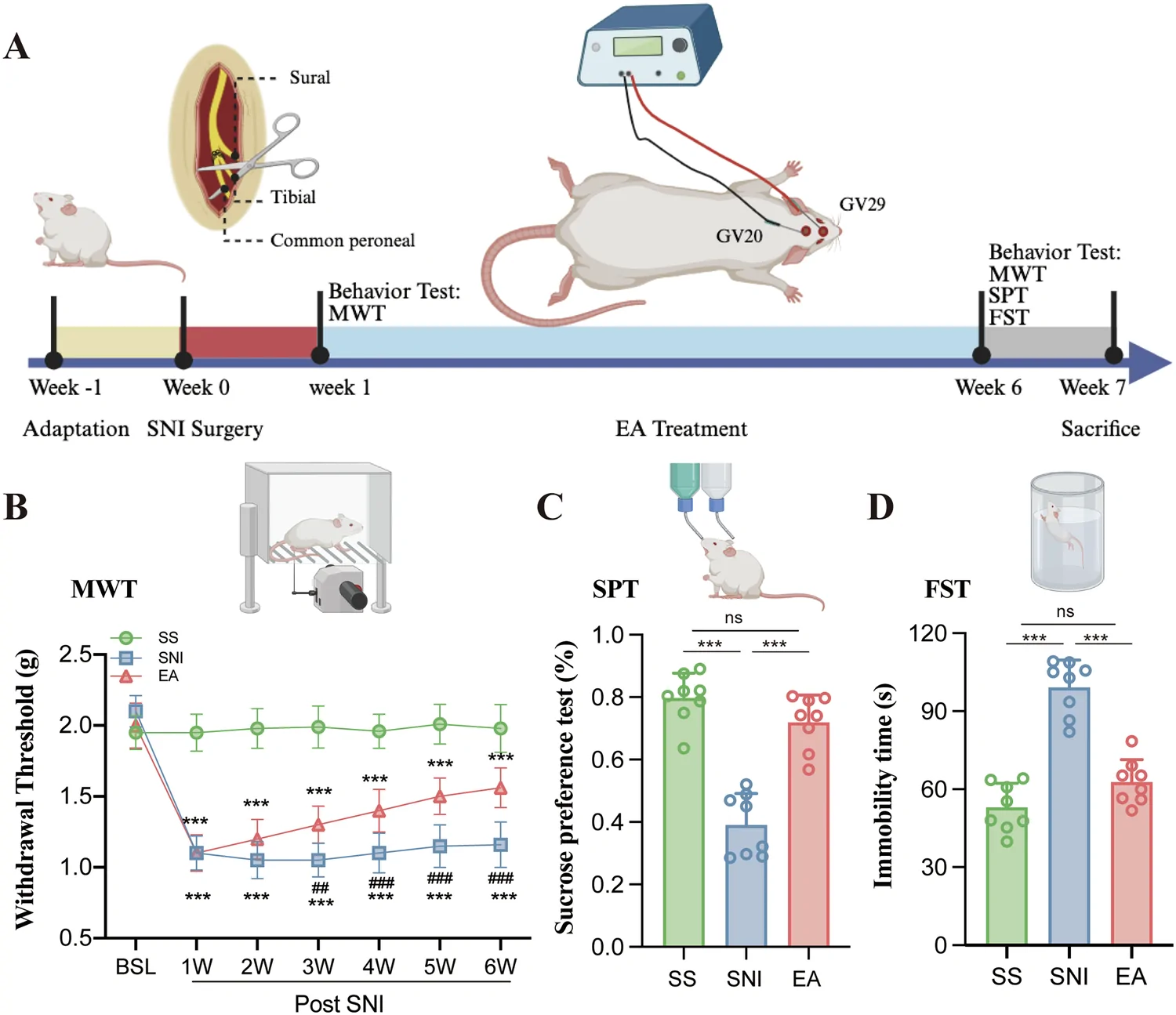
EA ameliorates SNI-induced pain and depression-like behaviors. **A** Schematic illustration of the experimental timeline. **B** Time course of the Mechanical Withdrawal Threshold. **C** Sucrose preference test. **D** Immobility time in forced swim test. N = 8 rat/group. Data are presented as mean ± SD. *P < 0.05, **P < 0.01, ***P < 0.001 vs. SS group; #*P* < 0.05, ##*P* < 0.01, ##*P* < 0.01 vs. EA group. Statistical comparisons were performed using Two-way repeated-measures ANOVA (for MWT) or One-way ANOVA (for SPT and FST) followed by Tukey’s post hoc test.

As depicted in Fig. 1A, following 1 week of acclimation, rats were randomly assigned to three groups (n = 8 per group) using a random number generator in SPSS software (version 25.0; IBM Corp., Armonk, NY, USA): the sham SNI group (SS), the SNI model group (SNI), and the EA group (EA). SNI surgery was performed on all rats in the SNI and EA groups, while rats in the SS group underwent a sham surgical operation. After a 1-week post-surgical recovery period, interventions were initiated. The EA group received EA treatment every other day for 5 weeks, while the SS and SNI groups were subjected to identical restraint and anesthesia in the absence of EA intervention. Investigators were not blinded during experiments and outcome assessment.

The Mechanical Withdrawal Threshold (MWT) was assessed 1 day prior to surgery (Baseline) and 1, 2, 3, 4, 5, and 6 weeks post-surgery. The Sucrose Preference Test (SPT) and the Forced Swim Test (FST) were performed 6 weeks after surgery.

### SNI surgery

The SNI surgery was performed as previously described [23]. Briefly, rats were anesthetized with 2.5% isoflurane, and an incision was made in the skin of the left thigh. The biceps femoris muscle was bluntly dissected to expose the sciatic nerve and its three terminal branches: the sural, common peroneal, and tibial nerves. The common peroneal and the tibial nerves were tightly ligated with 5.0 silk sutures and transected distal to the ligation. Muscle and skin were sutured in layers with 5.0 silk. Rats in the SS group underwent the same exposure procedure but without nerve ligation or transection.

### EA treatment

To minimize stress in rats, EA treatment was administered under light anesthesia with 1–1.5% isoflurane. Sterile acupuncture needles (0.18 mm × 15 mm; Hwato, Suzhou, China) were inserted at two acupuncture points: Baihui (GV20, located on the midsagittal line of the cranium, at the intersection of the sagittal midline and line connecting the apexes of the two auricles) and Yintang (GV29, located on the forehead midline, at the midpoint between the medial ends of the two eyebrows). An SDZ-III electronic acupuncture apparatus (Hwato, Suzhou, China) was employed to deliver EA stimulation at a frequency of 2 Hz for 30 min [24].

### Behavioral test

#### MWT

Mechanical allodynia was tracked using an automated Dynamic Plantar Aesthesiometer (DPA; Ugo Basile, Comerio, Italy). Prior to testing, rats were placed in individual Plexiglass chambers on a wire mesh floor and allowed to acclimatize for at least 30 min until exploratory behavior ceased. To measure the MWT, a rigid blunt filament (0.5 mm diameter) was automatically raised to stimulate the lateral part of the plantar surface of the left hind paw. The vertical force was increased gradually until a withdrawal reflex was elicited. The apparatus automatically recorded the force (in grams). To prevent tissue damage, a cut-off force of 50 g was enforced. Each rat was tested three times with an interval of 5 min between trials, and the average value was calculated as the MWT.

#### SPT

The SPT was performed to assess anhedonia. Briefly, on Day 1 (9:00), rats were individually housed and trained to consume 1% sucrose water (w/v) in two bottles. On Day 2, one sucrose bottle was replaced by pure water. On Day 3, rats were deprived of food and water for 24 h. On Day 4, rats were fed with two pre-weighed bottles: one containing 1% sucrose and the other containing pure water. On Day 5 (9:00), the two bottles were re-weighed. Sucrose preference was calculated as: Sucrose preference (%) = [sucrose consumption/ (sucrose consumption + water consumption)] × 100.

#### FST

The FST was conducted to evaluate behavioral despair. Rats were placed in a transparent cylindrical container (50 cm in height and 20 cm in diameter) filled with tap water (24 °C, 35 cm in depth) and forced to swim for 6 min [25]. The FST was performed in JLBehv-FSG-4 sound insulation boxes equipped with the Dig Behav animal behavior video analysis system (Shanghai Jiliang Software Technology Co. Ltd., Shanghai, China). The duration of immobility during the last 5 min of the total 6 min test was recorded. A rat was considered immobile when its head floated above the water and it ceased active movement.

### Tissue collection

Upon completing behavioral testing, rats were anesthetized with 1% sodium pentobarbital (i.p.). Blood was collected from the abdominal aorta into EDTA tubes, kept on ice, and centrifuged (3,000 × *g*, 15 min, 4 °C) to harvest plasma, which was aliquoted and stored at −80 °C. Fecal samples were directly collected from the distal colon, instantly frozen in liquid nitrogen, and stored at −80 °C for omics analysis. For central tissue isolation, the whole brain was rapidly transferred to an ice-cold brain matrix. Under the guidance of the Paxinos and Watson rat brain atlas, the medial prefrontal cortex (mPFC) was accurately microdissected (coordinates from Bregma: AP: +3.7 to +2.5 mm; ML: ±0.4 to ±0.8 mm; DV: −3.0 to −5.0 mm from the dura). The dissected mPFC tissue was immediately snap-frozen in liquid nitrogen and stored at −80 °C until further analysis.

### Fecal metagenomic sequencing and analysis

Bacterial genomic DNA was extracted from fecal samples using a cetyltrimethylammonium bromide method following the manufacturer’s instructions. DNA concentration and integrity were assessed to ensure sample quality for library construction. Sequencing libraries were generated using the NEBNext Ultra DNA Library Prep Kit (NEB, USA), and paired-end sequencing (2 × 150 bp) was performed on the Illumina NovaSeq 6000 platform. Raw reads were processed using Trimmomatic to remove low-quality sequences and adapters. To eliminate host contamination, clean reads were aligned to the Rattus norvegicus genome (e.g., Rnor_6.0) using Bowtie 2, retaining only non-host metagenomic sequences. Taxonomic classification was performed using Kraken 2, and species abundance was re-estimated using Bracken (Bayesian Reestimation of Abundance with KrakEN) at multiple phylogenetic levels (phylum to species). Sequencing and bioinformatics analyses were conducted by Wekemo Tech Group Co., Ltd. (Shenzhen, China).

### Plasma untargeted metabolomics analysis

Plasma samples (100 μL) were extracted with pre-chilled 80% methanol and centrifuged (14,000 × *g*, 20 min, 4 °C) to collect supernatants. Chromatographic separation was performed on a Vanquish UHPLC system using a Hypersil Gold column (100 × 2.1 mm, 1.9 μm; Thermo Fisher) with a 12-min linear gradient of 0.1% formic acid in water (A) and methanol (B) at 0.2 mL/min. Mass spectrometry was conducted on an Orbitrap Q Exactive™ HF/HF-X system (Thermo Fisher) in both positive and negative ionization modes under optimized parameters. Raw data processing, including peak alignment, picking, and normalization, was performed using Compound Discoverer 3.3 (Thermo Fisher). Metabolite identification relied on spectral matching against mzCloud, mzVault, and MassList databases. To ensure data quality, features with a coefficient of variation (CV) >30% in quality control (QC) samples were excluded.

### Enzyme-Linked Immunosorbent Assay (ELISA)

The absolute contents of 5-HT, NE, DA, GABA and Glu in the mPFC were quantified using rat ELISA kits (Cusabio, Wuhan, China) according to the manufacturer’s instructions. Briefly, approximately 15–20 mg of mPFC tissue per sample was rapidly micro-dissected and homogenized in 200 μL of ice-cold phosphate-buffered saline (PBS, pH 7.4) for 30 min. Then, the lysate was sonicated and clarified by centrifugation (12,000 × *g* for 20 min) at 4 °C. The supernatant extract was collected and quantified for total protein by using the BCA protein assay kit (Sigma Aldrich, St. Louis, MO, USA). Final concentration readouts were mathematically normalized against total protein content and expressed as picograms of target analyte per milligram of total protein (pg/mg protein). Absorbance was quantified using a microplate reader at 450 nm.

### Western Blotting (WB)

Total proteins were extracted from mPFC tissues using ice-cold RIPA lysis buffer (50 mM Tris, pH 7.4, 150 mM NaCl, 1% NP-40, 0.5% sodium deoxycholate, 0.1% SDS, and 1% PMSF) supplemented with a protease inhibitor cocktail and quantified as described previously. Equal amounts of protein were denatured at 100 °C for 10 min and separated by 10% SDS-PAGE (Beyotime, Shanghai, China). The proteins were subsequently transferred onto polyvinylidene difluoride membranes (Millipore, MA, USA) using wet transfer equipment (Bio-Rad, CA, USA). Membranes were blocked with 5% bovine serum albumin (Sigma-Aldrich, St. Louis, MO, USA) for 1 h at room temperature and then incubated overnight with primary antibodies at 4 °C. The membranes were washed three times in TBST (5 min each) and further incubated with horseradish peroxidase-conjugated secondary antibodies at room temperature for 1 h. Protein bands were visualized using enhanced chemiluminescence reagents (Millipore, MA, USA) and quantified with the ChemiDoc imaging system using Image Lab software (Bio-Rad, CA, USA). The primary antibodies used were specific to GAD67 (1:1000), EAAT2 (1:1000), and GAPDH (1:1000), all obtained from Cell Signaling Technology.

### Statistical analysis

All data are presented as the mean ± standard deviation (SD). Statistical analyses were performed using GraphPad Prism software (8.0.2) and RStudio (v4.3.0). Prior to parametric statistical testing for behavioral and molecular biological datasets, the normality of data distribution and homogeneity of variance were systematically assessed. Longitudinal MWT data were analyzed using a Two-way Repeated-Measures ANOVA with Time as the within-subject factor and Treatment as the between-subject factor, followed by Tukey’s post hoc multiple comparisons test. Comparisons between multiple groups were performed using a One-way ANOVA followed by Tukey’s post hoc test. A value of *P* < 0.05 was considered statistically significant. For multi-omics synthesis, Spearman’s rank correlation analysis was deployed to generate cross-biological layer correlation networks and heatmaps. To mathematically test directional causal chains and rule out non-causal parallel phenomena, a Nonparametric Bootstrap Causal Mediation Analysis (with 5000 simulations) was executed using the R mediation package.

## Results

### EA alleviates SNI-induced mechanical allodynia and depressive-like behaviors

To validate the therapeutic efficacy of EA, we first assessed the longitudinal nociceptive profiles alongside affective phenotypes. For the longitudinal pain assessment, Two-way Repeated-Measures ANOVA (configured with Geisser–Greenhouse correction for sphericity violations) performed on the MWT dataset revealed significant main effects of treatment [F(2, 21) = 68.77, *P* < 0.0001], time [F(5.261, 110.5) = 1251, *P* < 0.0001], and a treatment × time interaction [F(12, 126) = 410.3, *P* < 0.0001]. Post-hoc multiple comparisons demonstrated that SNI surgery induced a profound and immediate drop in mechanical thresholds starting from post-operative Week 1 (*P* < 0.0001 vs. SS), which was sustained throughout the 6-week post-operative timeline (Fig. 1B). Notably, EA intervention mitigated this mechanical hypersensitivity; a significant elevation in the MWT of the EA group was detected at Post-SNI Week 3 (*P* = 0.0036 vs. SNI) and steadily improved until the terminal assessment at Post-SNI Week 6 (P < 0.0001 vs. SNI), establishing a potent anti-allodynic action.

Concurrently, affective phenotypes evaluated at Post-SNI Week 6 revealed distinct depressive-like behaviors in the SNI group. In the SPT (Fig. 1C), the SNI group exhibited a marked reduction in sucrose preference compared to SS controls (*P* < 0.0001), which was significantly reversed by the EA regimen (*P* < 0.01 vs. SNI). In the FST (Fig. 1D), behavioral despair was evidenced by a prolonged immobility duration in the SNI group (*P* < 0.0001 vs. SS); EA intervention significantly reduced immobility time (*P* < 0.0001 vs. SNI), confirming a robust anti-depressive effect. Taken together, these behavioral data confirmed that EA comprehensively alleviates both the mechanical allodynia and depressive-like behaviors in the SNI-induced neuropathic pain model.

### EA restores gut microbial homeostasis and reshapes community structure

**Fig. 2:**
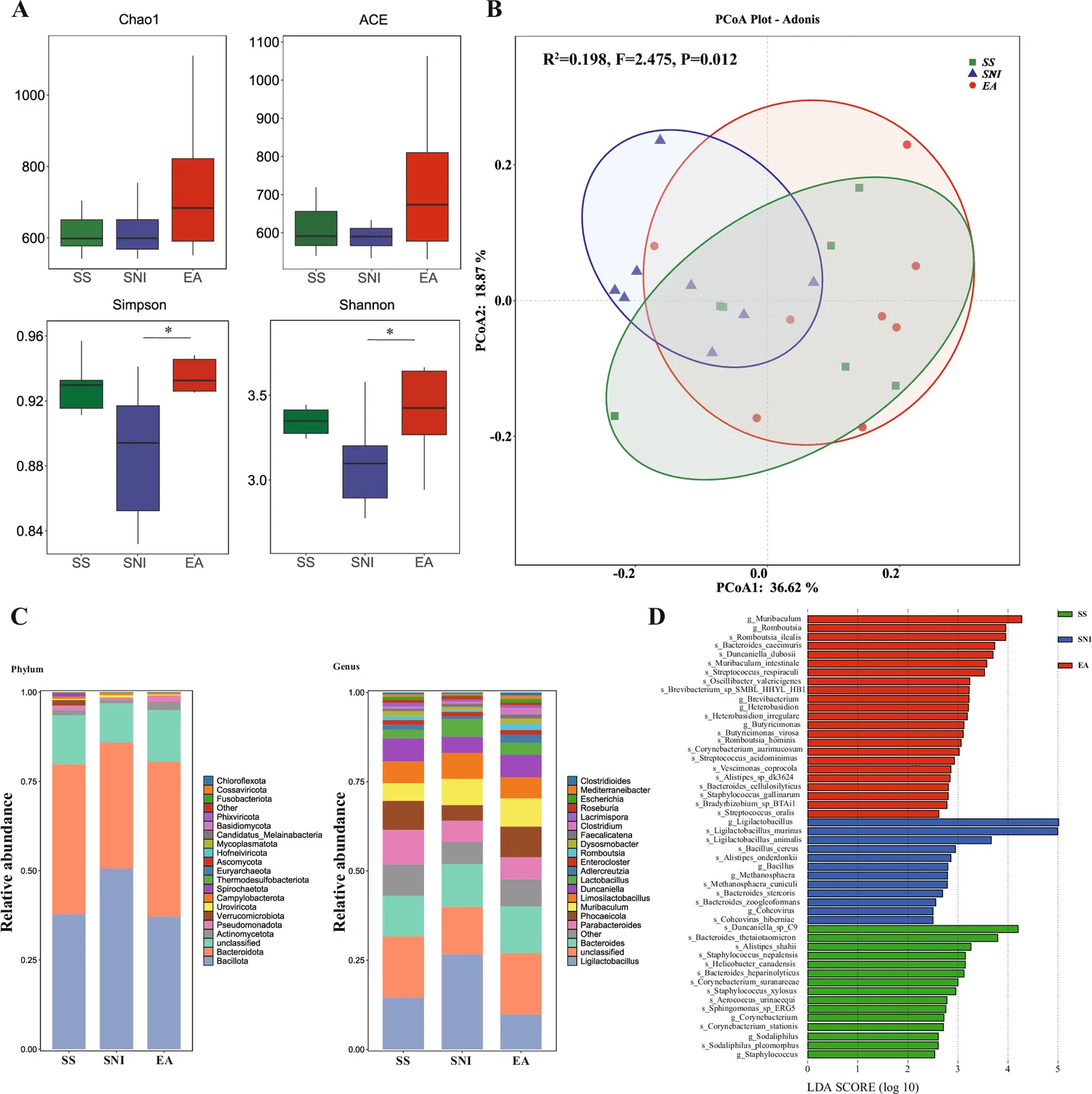
EA restores gut microbial homeostasis and reshapes community structure in SNI rats. **A** Alpha-diversity indices. **B** Beta-diversity analysis based on PCoA of Bray-Curtis distances. **C** Relative abundance of gut microbiota at the phylum and genus levels. **D** Differentially abundant taxa identified by LEfSe. The histogram shows LDA scores (>2.5) of distinct taxa at the genus and species levels. N = 7–8 rat/group. Statistical significance for alpha-diversity was determined using the Kruskal-Wallis test followed by the Wilcoxon rank-sum test. Beta-diversity differences were assessed using Adonis (PERMANOVA) analysis.

Metagenomic sequencing was employed to decipher the gut microbial alterations underlying EA efficacy. As shown in Fig. 2A, while species richness (ACE and Chao1 indices) showed no significant inter-group differences, EA treatment significantly restored microbial evenness—a key indicator of ecological stability. Specifically, both the Shannon (*P* = 0.038) and Simpson (*P* = 0.021) indices were significantly elevated in the EA group compared to SNI rats, suggesting re-establishment of a more balanced and stable microbial ecosystem. Beta diversity analysis via Principal Coordinates Analysis (PCoA) confirmed distinct community structures among groups (Adonis, *P* = 0.012, Fig. 2B), with EA treatment shifting the microbial profile away from the SNI pattern.

Discriminative analysis (LEfSe) further pinpointed specific taxa driving these shifts (LDA score >2.5, *P* < 0.05; Fig. 2D). The SNI phenotype was associated with an expansion of pro-inflammatory bacteria taxa including *Bacillus cereus*, *Alistipes onderdonkii, and Methanosphaera cuniculi*. Conversely, the EA group was significantly enriched with SCFA-associated beneficial commensals, including *Butyricimonas, Romboutsia, Bacteroides caecimuris, and Oscillibacter valericigenes*. These structural shifts indicated that EA treatment selectively promotes the abundance of specific commensals associated with metabolic homeostasis.

### EA modulates systemic metabolic profiles and rebalances mPFC neurotransmission

**Fig. 3:**
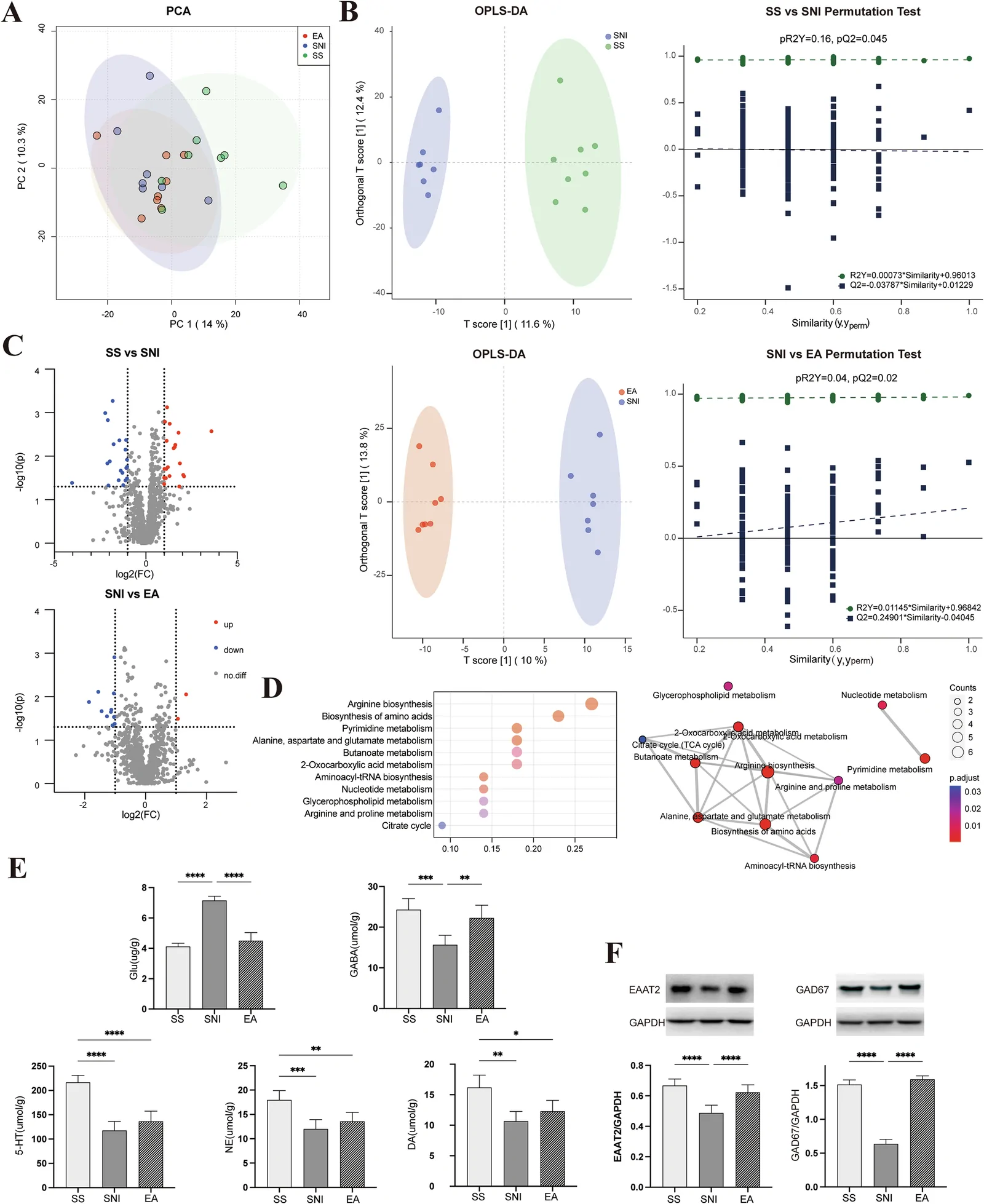
EA modulates systemic metabolic profiles and rebalances PFC neurotransmission. **A** PCA score plot of plasma metabolites from the SS, SNI, and EA groups. **B** OPLS-DA score plot and permutation test validation. **C** Volcano plot illustrating differentially abundant metabolites. Red dots indicate upregulated metabolites, and blue dots indicate downregulated metabolites. **D** KEGG pathway enrichment analysis of differential metabolites for EA vs. SNI groups. **E** Levels of neurotransmitters (5-HT, NE, DA, Glu, GABA) in the PFC measured by ELISA. Data are presented as mean ± SD (n = 7–8 rats/group). Statistical significance indicated by *P < 0.05, **P < 0.01, ***P < 0.001.

To link gut dysbiosis to central outcomes, we profiled the plasma metabolome using untargeted LC-MS/MS metabolomics. Principal Component Analysis (PCA; Fig. 3A and B) revealed distinct clustering patterns, with the SS group markedly separated from the SNI and EA groups, indicating robust metabolic perturbations induced by SNI. Orthogonal Partial Least Squares Discriminant Analysis (OPLS-DA) further delineated the metabolic signatures, showing clear separation among groups with no evidence of overfitting (permutation test). A total of 1,084 metabolites were detected, of which 21 were significantly upregulated and 20 were significantly downregulated in the SNI group compared to the SS group, based on the criteria of *P* < 0.05 and |log2FC| >1 (Fig. 3C). In contrast, the EA group exhibited 11 significantly downregulated and 2 significantly upregulated metabolites compared to the SNI group, indicating a modulatory effect of EA on SNI-induced metabolic alterations.

Kyoto Encyclopedia of Genes and Genomes (KEGG) pathway overrepresentation analysis (ORA) was conducted using the cluster Profiler package in R. ORA identified 11 significantly enriched KEGG pathways (Fig. 3D), including amino acid biosynthesis and metabolism, with particular emphasis on neurotransmitter-related amino acid metabolism (including glutamate, arginine, and associated pathways), alongside butanoate metabolism.

Consistent with these systemic metabolic shifts, we quantified neurotransmitter levels in the PFC (Fig. 3E). The SNI group displayed a generalized collapse of monoaminergic and GABAergic tone, characterized by reduced levels of 5-HT, NE, DA, and GABA, concurrent with excitotoxic elevation of Glu. Importantly, EA treatment significantly rectified the Glu/GABA imbalance, specifically by reversing the aberrant levels of Glu and GABA (*P* < 0.01).

To further validate the molecular mechanisms underlying the restoration of E/I balance, we examined the expression of GAD67 (the rate-limiting enzyme for GABA synthesis) and EAAT2 (the primary astrocytic glutamate transporter) in the mPFC (Fig. 3F). The SNI group exhibited a severe downregulation of both GAD67 (*P* < 0.01) and EAAT2 (*P* < 0.01) expression compared with the SS group. Strikingly, the EA intervention significantly upregulated the expression of both GAD67 (*P* = 0.0028) and EAAT2 (*P* = 0.0015), underscoring its role in regulating critical enzymatic and transport machinery required to maintain Glu/GABA homeostasis.

### EA drives systemic metabolic remodeling to normalize mPFC Glu/GABA balance and reverse co-morbid pain and depressive behaviors

**Fig. 4:**
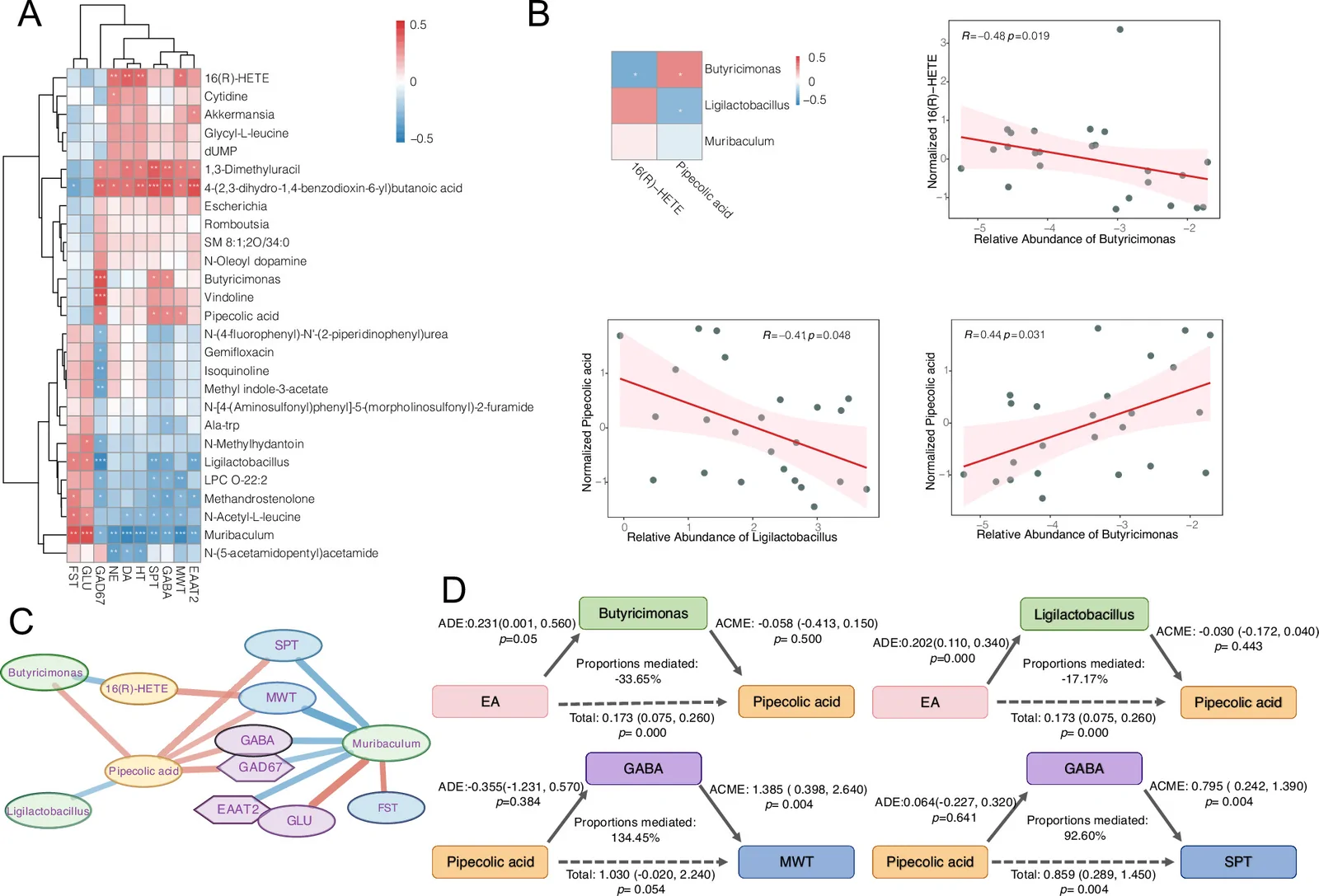
Integrative multi-omics analyses identify a gut–metabolite–brain network associated with mPFC neurotransmission and behavioral recovery following electroacupuncture. **A** Integrative correlation heatmap illustrating coordinated associations among gut microbial alterations, plasma metabolites, mPFC neurochemical markers, and behavioral outcomes. Asterisks indicate statistically significant correlations. **B** Correlation analysis between EA-responsive gut microbial taxa and differential plasma metabolites, presented as a correlation heatmap and representative scatter plots. **C** Integrative network analysis identifies Pipecolic acid as a central metabolic node linking gut microbial alterations with mPFC neurotransmission and behavioral recovery. **D** Causal mediation analysis identifies the Pipecolic acid–mPFC GABA pathway as a significant mediator associated with improvements in mechanical allodynia and anhedonia following EA treatment.

To construct a functional bridge spanning the enteric micro-ecosystem, peripheral circulation, and central neuro-behavioral phenotypes, we performed an integrated multi-omics association network analysis. Candidate multi-omics features were strictly filtered based on rigorous statistical thresholds. Specifically, gut microbiota taxa were selected if they exhibited significant tripartite alterations (Kruskal–Wallis *P* < 0.05) or served as primary taxonomical discriminators (LEfSe LDA score >2). Concurrently, plasma metabolites were integrated only if they met the selection criteria of exhibiting therapeutic responsiveness during EA intervention, defined as showing significant multi-group statistical variance between the SNI and EA cohorts, alongside an absolute magnitude of change |log2 FC| >0.5 and VIP >1 in the OPLS-DA model. Inter-systemic Spearman’s rank correlation matrices revealed that three specific bacterial genera (*Butyricimonas*, *Ligilactobacillus*, and *Muribaculum*) and seven plasma metabolites were tightly coupled with prefrontal neurotransmitter pools and the somatosensory and affective behavioral outcomes (Fig. 4A).

To further clarify how these micro-ecological alterations were linked with circulating metabolic shifts, we evaluated the correlation among these candidate genera and the KEGG pathway-associated metabolites. Strikingly, the EA-enriched genus *Butyricimonas* displayed powerful, coordinated correlations with two pivotal functional intermediates (Fig. 4B): mapping positively to plasma Pipecolic acid (R = 0.44, *P* = 0.031), a major L-lysine catabolic product with high blood-brain permeability, and negatively to 16(R)-HETE (R = −0.48, *P* = 0.019), an arachidonic acid-derived lipid mediator. Conversely, *Ligilactobacillus* exhibited a distinct negative association with the circulating Pipecolic acid flux (R = −0.41, *P* = 0.048).

Next, we integrated these multi-omics features and behavioral phenotypes into a comprehensive topology (Fig. 4C) to visualize the gut–metabolite–brain axis. Strikingly, Pipecolic acid emerged as the central strategic hub. It linked upstream microbial shifts (*Butyricimonas*, *Ligilactobacillus*) to the mPFC GABAergic machinery, mapping tightly onto the alleviation of mechanical allodynia and depressive-like behaviors.

To further evaluate the functional Gut-Metabolite-Brain axis suggested by our multi-omics network, we performed bootstrap mediation analysis (5000 simulations). EA, individual bacterial genera and circulating Pipecolic acid did not show significant mediation effects (*P* > 0.05), suggesting that EA-induced metabolic remodeling is unlikely to be explained by alterations in any single bacterial genus but rather reflects coordinated microbial ecosystem remodeling. In contrast, a significant indirect pathway was identified between circulating Pipecolic acid, mPFC GABA and behavioral outcomes. Specifically, GABA significantly mediated the association between Pipecolic acid and mechanical sensitivity (ACME = 1.3845, *P* = 0.004), whereas the direct effect was not statistically significant. A similar mediation pattern was observed for depressive-like behavior, with GABA significantly mediating the association between Pipecolic acid and sucrose preference (ACME = 0.7954, *P* = 0.004), accounting for approximately 92.6% of the total effect (Fig. 4D). Collectively, these findings provide quantitative support for a functional Gut-Metabolite-Brain pathway in which peripheral metabolic remodeling is closely associated with central GABAergic neurotransmission and behavioral recovery following EA treatment.

## Discussion

In the present study, we demonstrate that EA effectively ameliorates the comorbidity of neuropathic pain and depression in the SNI rat model. By integrating fecal metagenomics and plasma untargeted metabolomics, we reveal that the therapeutic benefits of EA are inextricably linked to the systemic restoration of the “microbiota–gut–brain axis”. Specifically, EA reshaped the dysbiotic gut microbial ecosystem by increasing microbial diversity and suppressing opportunistic pathobionts, reprogrammed systemic metabolism toward amino acid biosynthesis and butanoate metabolism, restored mPFC Glu/GABA homeostasis, and consequently improved both pain- and depression-like behaviors. Importantly, our integrative analyses extend these observations beyond descriptive multi-omics profiling by identifying Pipecolic acid as a central metabolic node linking gut microbial remodeling with mPFC GABAergic neurotransmission and behavioral recovery. This finding shifts the mechanistic emphasis from individual microbial taxa toward the coordinated functional metabolic outputs of the microbial ecosystem, suggesting that the therapeutic actions of EA are more closely associated with restoration of gut-derived metabolic homeostasis than with alterations in any single bacterial species.

### EA restores mPFC Glu/GABA homeostasis to improve pain–depression comorbidity

In this study, the EA intervention concurrently alleviated mechanical hypersensitivity and depressive-like behaviors in SNI rats, consistent with our previous findings and highlighting its significant therapeutic potential for managing pain–affective comorbidities [22]. Crucially, these behavioral improvements were accompanied by the restoration of glutamate/GABA homeostasis by modulating the expression of EAAT2 and GAD67 in the mPFC, suggesting that the homeostatic restoration of synaptic Glu/GABA balance is closely linked to the behavioral improvements elicited by EA. Growing literature further indicates that the collaborative regulation of glutamatergic and GABAergic neurotransmission represents a core, conserved neurochemical response to acupuncture in pain-associated affective comorbidities [26, 27]. However, these central neuromodulatory effects should not be evaluated in isolation. As a wealth of evidence suggests, gut-derived microbial and metabolic signals actively gate and fine-tune neuro-functional programs in the brain through the bidirectional microbiota–gut–brain axis [12, 16].

### EA remodels gut microbial ecology

EA substantially remodeled the gut microbial ecosystem disrupted by SNI. Specifically, EA restored microbial community organization by increasing α-diversity, suppressing opportunistic pathobionts, including Bacillus cereus, Alistipes onderdonkii, and Methanosphaera cuniculi, while enriching multiple metabolically beneficial genera associated with SCFA production, intestinal barrier maintenance, and host metabolic homeostasis, including Butyricimonas, Ligilactobacillus, and Muribaculum [28, 29]. Together, these findings indicate that EA re-establishes a gut microbial community associated with improved intestinal and metabolic homeostasis, providing a microbial basis for its systemic therapeutic effects. Importantly, correlation-based multi-omics network analysis demonstrated that several EA-responsive bacterial genera, including *Butyricimonas, Ligilactobacillus*, and *Muribaculum*, were associated with mPFC neurotransmitter levels and behavioral outcomes, indicating coordinated interactions across the gut–metabolite–brain axis. However, mediation analysis did not identify a significant indirect effect of either *Butyricimonas* or *Ligilactobacillus* on plasma Pipecolic acid, indicating that the observed microbial–host interactions cannot be adequately explained by individual bacterial genera alone. These findings are consistent with the emerging concept that gut microbiota regulates host physiology primarily through community-level ecological interactions and coordinated metabolic functions rather than through single microbial species [30, 31]. Accordingly, our integrative analyses suggest that the therapeutic effects of EA are more likely to arise from functional reorganization of the gut microbial ecosystem and its collective metabolic output than from alterations in individual bacterial taxa. Mechanistically, these ecological shifts may be influenced by a neuro-visceral regulatory framework, whereby EA-induced activation of somatic sensory pathways engages central autonomic and neuroendocrine networks [19, 32]. These descending regulatory processes may modulate systemic inflammatory tone and intestinal barrier function, thereby creating a permissive peripheral environment for microbial ecological recovery [33].

### Peripheral metabolic remodeling functionally bridges gut microbial ecology and central neurotransmission

Beyond remodeling the gut microbial ecosystem, EA coordinately remodeled host metabolism, particularly amino acid and butanoate metabolism, two metabolic programs increasingly recognized as key biochemical interfaces between the gut microbiota and the central nervous system [15, 34]. These pathways provide the metabolic basis through which microbial activity influences neurotransmitter synthesis, neuroimmune regulation, and neuronal plasticity, suggesting that the metabolic alterations induced by EA represent functional consequences of microbial ecosystem remodeling rather than isolated changes in circulating metabolites. Increasing evidence indicates that microbiota–gut–brain communication is mediated primarily by circulating neuroactive metabolites rather than individual microbial taxa [12, 35]. Within this framework, Pipecolic acid, a byproduct of lysine metabolism that has been linked to GABAergic signaling and neurotransmitter release [36], emerged as a potential functional metabolic hub linking gut microbial metabolism with mPFC neurochemical regulation. Supporting this interpretation, our integrative analyses showed that Pipecolic acid occupied a central position in the multi-omics network connecting EA-responsive bacterial genera with mPFC GABA levels and behavioral phenotypes, whereas mediation analysis identified the Pipecolic acid–GABA–behavior axis, rather than individual bacterial genera, as the principal pathway associated with behavioral recovery. Although microbiota–gut–brain communication is inherently bidirectional, these findings support a model in which EA-induced microbial ecosystem reorganization converges on functionally relevant circulating metabolites, with Pipecolic acid representing a plausible metabolic hub linking peripheral metabolic remodeling to restoration of mPFC GABAergic neurotransmission.

This study has several limitations. First, although integrative multi-omics analyses and mediation analyses support the proposed gut–metabolite–brain pathway, direct functional validation remains necessary. Future studies employing targeted manipulation of Pipecolic acid and microbiota transplantation or depletion strategies will be required to further clarify the causal relationships within this axis. Second, the neurochemical assessment was restricted to the mPFC; future studies should investigate additional brain regions involved in pain–depression comorbidity. Third, whether the observed gut–metabolite–brain effects are dependent on specific acupoint combinations or stimulation parameters remains unclear, warranting systematic comparison of different EA protocols. Finally, only male rats were included; future studies incorporating both sexes and clinical cohorts are needed to further evaluate the generalizability and translational relevance.

## Conclusion

In summary, our study provides integrated multi-omics evidence that EA alleviates SNI-induced pain and depression through remodeling of the Gut-Metabolite-Brain axis. EA induced coordinated reconstruction of the gut microbial ecosystem and peripheral metabolic remodeling, which was functionally associated with restoration of mPFC Glu/GABA balance and behavioral recovery. Integrative multi-omics analyses further identified Pipecolic acid as a potential functional metabolic hub linking peripheral metabolic remodeling with central inhibitory neurotransmission. Collectively, these findings provide a systems-level framework for understanding the therapeutic mechanisms of EA and highlight peripheral metabolic remodeling as a promising translational target for pain–depression comorbidity.

## Data Availability

Raw metagenomic sequencing data have been deposited in the Genome Sequence Archive (GSA) under the BioProject accession number PRJCA076187. Metabolomics data and other supporting data are available from the corresponding author upon reasonable request.
